# The Incidence, Electrophysiological Characteristics and Ablation Outcome of Left Atrial Tachycardias after Pulmonary Vein Isolation Using Three Different Ablation Technologies

**DOI:** 10.3390/jcdd9020050

**Published:** 2022-02-03

**Authors:** Patrick Leitz, Kristina Wasmer, Christian Andresen, Fatih Güner, Julia Köbe, Benjamin Rath, Florian Reinke, Julian Wolfes, Philipp S. Lange, Christian Ellermann, Gerrit Frommeyer, Lars Eckardt

**Affiliations:** Department of Cardiology II—Electrophysiology, University Hospital Muenster, Cardiol, Albert-Schweitzer-Campus 1, Building A1, 48149 Muenster, Germany; wasmerk@ukmuenster.de (K.W.); christian.andresen@ukmuenster.de (C.A.); fatih.guener@ukmuenster.de (F.G.); julia.koebe@ukmuenster.de (J.K.); benjamin.rath@ukmuenster.de (B.R.); florian.reinke@ukmuenster.de (F.R.); julian.wolfes@ukmuenster.de (J.W.); philippsebastian.lange@ukmuenster.de (P.S.L.); christian.ellermann@ukmuenster.de (C.E.); gerrit.frommeyer@ukmuenster.de (G.F.); lars.eckardt@ukmuenster.de (L.E.)

**Keywords:** pulmonary vein isolation, atrial fibrillation, left atrial flutter

## Abstract

Background: Left atrial tachycardias (LAT) are a well-known outcome of pulmonary vein isolation (PVI). Few data are available on whether the catheter used to perform PVI influences the incidence, as well as the characteristics of post PVI LAT. We present data on LAT following PVI by the following three ablation technologies: (1) phased multi-electrode radiofrequency catheter (PVAC), (2) irrigated single-tip catheter (iRF), and (3) cryoballoon ablation. Methods: Using a prospectively designed single-center database, we analyzed 650 patients (300 iRF, 150 PVAC, and 200 cryoballoon) with paroxysmal (*n* = 401) and persistent atrial fibrillation (AF), who underwent their first PVI at our center. Results: The three populations were comparable in their baseline characteristics; however, the cryoballoon group comprised a higher percentage of patients with persistent AF (*p* = 0.05). The LAT rates were 3.7% in the iRF group (mean follow-up 22 ± 14 months), 0.7% in the PVAC group (mean follow-up 21 ± 14 months), and 4% in the cryoballoon group (mean follow-up 15 ± 8 months). The predominant mechanism of LAT was macro-reentrant tachycardia. Reconnection of at least one pulmonary vein was observed in 87% of the patients who underwent 3D mapping. No predictors for LAT occurrence were identified. Conclusion: The occurrence of LAT post PVI is rare; the predominant mechanism was macro-reentrant tachycardia. Reconnection of at least one pulmonary vein was observed in nearly all the LAT patients. In our retrospective analysis, the lowest rate of LAT was observed with the PVAC. No predictors for LAT occurrence were identified.

## 1. Introduction

Atrial fibrillation (AF) is the most widespread sustained arrhythmia [1] and a major cause of stroke [2]. In symptomatic patients, pulmonary vein isolation (PVI) has steadily gained importance in comparison to antiarrhythmic drugs [3,4]. Developments such as the cryoballoon and the multipolar phased ablation catheter have allowed for shorter procedural times with comparable efficacy and safety as the established single-tip point-by-point ablation [5,6].

A well-known outcome of PVI is the occurrence of left atrial tachycardias (LAT), which are often more symptomatic than the initial AF. It remains unclear if the technology used for PVI affects the occurrence rates and electrophysiological mechanisms of post PVI LAT. We sought to determine the occurrence rates, as well as mechanisms, of post PVI LAT after ablation, performed with the following three technical approaches: (1) single-tip irrigated RF catheter (iRF), (2) phased multipolar RF ablation catheter (PVAC), and (3) a cryoballoon system.

## 2. Methods

### 2.1. Study Design and Patient Collective

We included a total of 650 patients who were referred to our center for an index PVI. Data were collected from a prospectively designed database, as well as our outpatient program. Patients were seen three months after PVI for an in-person visit. After 6 and 12 months, telephone interviews were performed. Further telephone interviews were scheduled in 6–12-month intervals. Additionally, patients were instructed to present to our department in case of symptomatic arrhythmias. Data collection was performed from 2011 to 2020. Exclusion criteria were defined as follows: severe valvular disease, prior surgical- or catheter-based PVI, prior linear ablations, inability to give informed consent, pregnancy, <18 years old.

### 2.2. Diagnosis of LAT

During the in-person visits and telephone interviews, patients were interrogated on arrhythmia occurrence. In the case of recurrence, ECG documentation was obtained. LATs within 3 months following PVI were discarded from the analysis.

### 2.3. Index PVI

The catheter used for PVI was chosen at the operator’s discretion. Point-by-point RF ablation (St. Jude Medical, Saint Paul, MN, USA), ablation with the PVAC (Medtronic Inc., Minneapolis, MN, USA) system, as well as the cryoballoon (Medtronic Inc., Minneapolis, MN, USA) performed at our center have been described in detail in previous publications [7,8,9,10]. In brief, the groups are outlined below:

(1) iRF group: iRF ablation was guided by the NAVX system (Ensite NavXVelocity; St. Jude Medical, Inc., Saint Paul, MN, USA). A 3D model was created with a circular mapping catheter (Inquiry Optima; St. Jude Medical Inc.), and the acquired geometry was fused with the 3D reconstruction of the LA from computed tomography (CT). Ablation was performed with a 4 mm open-tip irrigated catheter (IBI TherapyCoolpath Duo or CoolFlex; St. Jude Medical, Inc). Antral point-by-point circumferential ablation around ipsilateral PVs, with a distance from 0.5 to 1.0 cm from the ostia, was performed. The power was set to 30 W and the temperature was limited to 43 °C. In case of persistent PV conduction, especially at the anterior ridge border of the lateral PVs, we increased the power to 35 W, to close conduction gaps. Complete electrical isolation was monitored and confirmed by the circular mapping catheter during sinus rhythm and differential pacing maneuvers. Ablation lesions were limited to circumferential PVI.
(2) PVAC group: Ablation with the PVAC system was performed using the second-generation catheter, the PVAC GOLD (Medtronic Inc., Minneapolis, MN, USA). The PVAC was connected to the GENius (Medtronic Inc.) RF generator. The target temperature was 60 °C, with a maximum power of 8 W. Duty-cycled energy delivery was exclusively delivered in a 4:1 ratio, and the impulse duration was 60 s. Electrode pairs with insufficient tissue contact were selectively deactivated. After RF applications, the PVAC was rotated around the PV ostium, looking for the earliest pulmonary vein potential (PVP) to completely isolate the vein. Finally, the catheter electrodes were advanced in the PV demonstrating the entrance block; the exit block was not routinely checked for. No other catheters were used for touch-up lesions. No further ablations were performed.
(3) Cryoballoon group: All cryoballoon patients were treated with the second-generation 28 mm cryoballoon (Medtronic Arctic Front Advance, Medtronic Inc.). Occlusion of the PV was tested by a contrasting agent. In this cohort, 2 freezes were applied for 180 s per vein, irrespective of the achieved time to isolation (TTI). Additional freezes were applied if PVI was not achieved. Energy delivery was prematurely stopped in the case of inadequate temperature development, or if PVI could not be achieved within the first 90 s of the freeze. Ablation of the right PVs was performed under continuous stimulation of the phrenic nerve with a cycle length of 800 ms. Freezes were immediately stopped if temperatures fell below −60 °C, or if phrenic nerve capture was lost. Complete electrical isolation of the PVs was confirmed using the Achieve mapping catheter in sinus rhythm and during differential pacing maneuvers. At the end of the procedure, PVI was verified in sinus rhythm. No further linear ablations were performed and no supplementary ablation catheters were used.

### 2.4. Ablation Procedures of Post PVI Left Atrial Tachycardia

Mapping of LAT was performed using either NAVX (St. Jude Medical, Saint Paul, MN, USA) or CARTO (Biosense Webster, Diamond Bar, CA, USA) systems. In the more recent cases, multielectrode high-density catheters were used (Pentaray for CARTO, HD Grid Catheter for NAVX). The ablation catheter was either a 3.5 mm irrigated-tip ablation catheter (NAVISTAR Thermo-Cool, Biosense-Webster) or a 4 mm irrigated-tip ablation catheter (IBI Therapy Coolpath Duo 7F, St. Jude Medical, Inc.). In more recent cases, contact force sensing irrigated-tip catheters were used (ThermoCool SmartTouch, Biosense-Webster/TactiCath, Sensor Enabled, St. Jude Medical).

A 3-dimensional activation and voltage map was acquired in all patients. The LATs were characterized upon their activation pattern, the presence of an “early meets late” region, and the differential pacing maneuvers (see video of exemplary activation map of peri-mitral and roof-dependent flutters in Supplementary material).

RF ablation was performed at a power of 30 W (maximum 25 W in the coronary sinus and maximum 40 W in the LA, in the case of persistent conduction gaps). Peri-mitral flutter was usually treated by an anterior mitral isthmus line. Upon termination of LAT, conduction block of linear lesions was checked through differential pacing and activation re-mapping. Programmed atrial stimulation was performed in order to reinduce the atrial arrhythmia. Finally, in the case of PV reconnection, re-isolation was performed using the same RF catheter used for LAT ablation.

### 2.5. Statistical Analysis

Statistical analysis was performed using SPSS (version 25, IBM SPSS Statistics, Armonk, NY, USA). Continuous data were expressed as mean ± SD and compared between groups using a Mann–Whitney U test. Categorical data were summarized by their observed frequencies and percentages, and compared using cross tabulation and a chi-Square test. For all statistical tests, a value of *p* < 0.05 was considered significant.

## 3. Results

### 3.1. Population Characteristics and Antiarrhythmic Drug Treatment

A total of 650 patients (mean age 61 years old, 36% persistent AF) were included in the study. The three groups were comparable in baseline characteristics (Table 1).

All the patients were advised to stop all antiarrhythmic drugs 3 months after the index PVI. At the time of LAT ablation, the PVAC patient presented with flecainide. In the iRF group, one patient was on amiodarone, three were on dronedarone, and four were on flecainide/propafenone. In the cryoballoon group, three patients were on amiodarone at the time of LAT ablation.

### 3.2. Occurrence and Characteristics of LATs

During a mean follow-up of 19 ± 8 months, 20 patients developed LAT. Three patients declined an electrophysiological study and opted for an electrical cardioversion. The diagnosis of LAT in these three patients was made based on the ECG, which was reviewed by at least two experienced electrophysiologists. In two patients, LAT terminated during or prior to 3D mapping; therefore, the LAT diagnosis was made through differential pacing.

Of the 150 PVAC patients, 1 patient (0.7%; paroxysmal AF; 48 years old; LAVI 40 mL/m^2^; 2 PVs reconnected) developed peri-mitral macro-reentry during a mean follow-up of 21 ± 14 months. In the iRF group, 11 of the 300 patients included (3.7%; 4 with persistent AF; mean age 63 years; mean LAVI 38.4 mL/m^2^; at least one PV reconnected in 81%) developed LAT during a mean follow-up of 22 ± 14 months. In the cryoballoon group, eight LAT patients developed LAT over a mean follow-up of 15 ± 8 months (4%; six males, five with persistent AF; mean age 62 ± 12 years; at least one PV reconnection in 63%).

**Figure 1:**
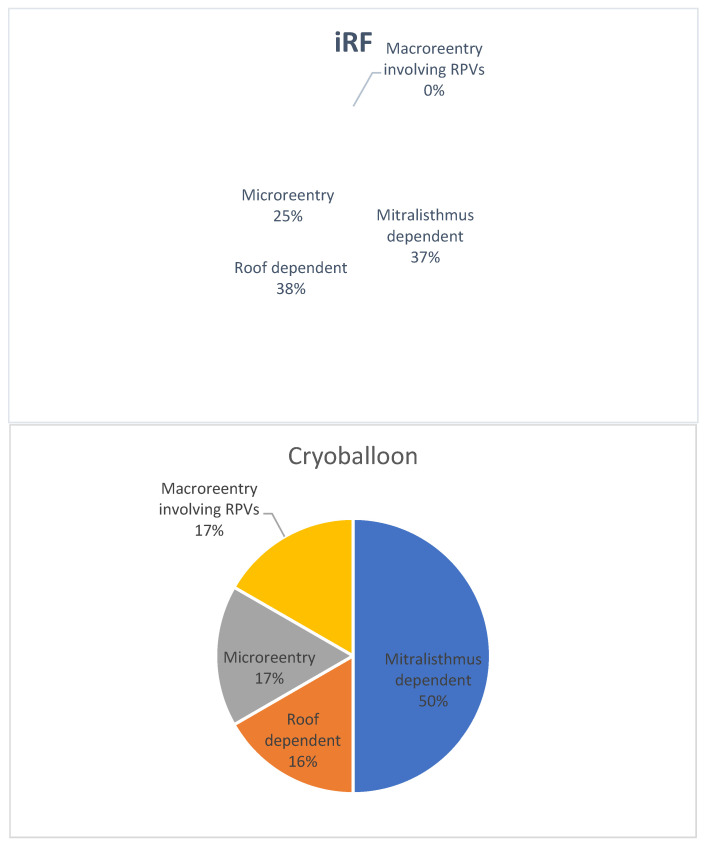

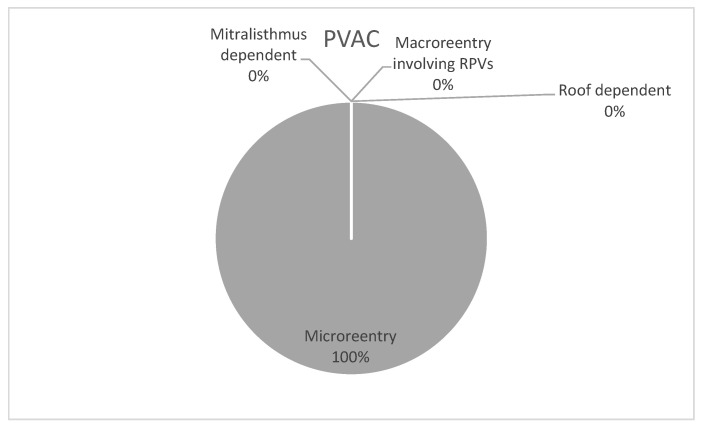
Mechanisms of LAT following PVI (RPV: right pulmonary veins).

**Figure 2:**
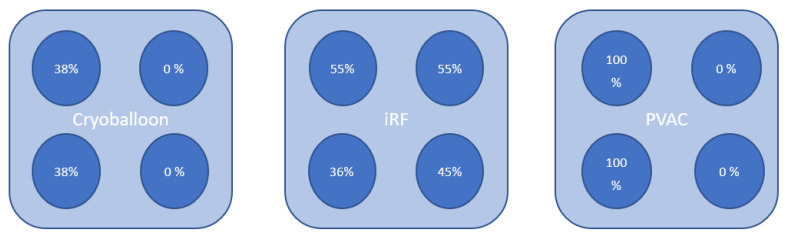
Schematic left anterior oblique view of the LA with the percentage of PV reconnections in the LAT patients.

The LAT cycle lengths (CL) ranged between 220 and 370 ms, with a mean of 252 ± 36 ms. Forty-five percent of the LAT patients had persistent AF at the time of the index PVI, and the mean left atrial volume (LAVI) was 40 ± 11 mL/m^2^ (see Table 2 and Figure 1 for the detailed characteristics and comparative occurrence rates of different LAT mechanisms). Figure 2 shows the percentages of PV reconnections in the LAT patients.

### 3.3. Possible Predictors of LAT

We performed a logistic univariate analysis to screen for predictors of LAT after PVI (see Table 3). Considering the entire study population, neither the age at the time of the index procedure (*p* = 0.76), CHA_2_DS_2_-Vasc (*p* = 0.1), male gender (*p* = 0.90), the presence of non-ischemic cardiomyopathy (NICM) (*p* = 0.39), the presence of ischemic cardiomyopathy (ICM) (*p* = 0.43), nor the left ventricular ejection fraction (LVEF) (*p* = 0.44) showed a significant correlation with the occurrence of LAT. Further, none of these characteristics reached statistical significance for predicting LAT occurrence in the different subgroups (iRF, cryoballoon and PVAC). For the cryoballoon group, the total freeze duration (*p* = 0.83), total number of freezes (*p* = 0.3), time to isolation (TTI) recorded in LSPV (*p* = 0.73), TTI recorded in RIPV (*p*= 0.09), and TTI recorded in RSPV (*p* = 0.63) showed no significant correlation with LAT occurrence. In the iRF and PVAC groups, the total RF durations also failed to reach statistical significance (*p* = 0.5 resp. *p* = 0.34). 

## 4. Discussion

A direct comparison of the three different catheter technologies used for PVI ablation, in terms of their LAT rates, has, to the best of our knowledge, not yet been published. In the entire cohort, we were able to identify an occurrence of LAT of 3%. The lowest rate of LAT was observed with phased RF technology, followed by comparable LAT rates for point-by-point RF ablation and cryoballoon technology. No independent predictors of LAT occurrence were identified. Nearly all the patients with LAT showed mild-to-severe LA dilation, a well-known marker for advanced atrial electrical and structural remodeling [11]. The majority presented with paroxysmal AF at the time of the index PVI. There was no apparent difference in the mechanisms observed, although, in the PVAC group, only one patient developed LAT during follow up, which allows no valid comparison. Macro-reentry was the predominant mechanism of LAT. In most cases, the reentry was LA roof or mitralisthmus dependent. Of note, in the patients with macro-reentry LAT, reconnection of at least one pulmonary vein was observed in most cases. In the patients with micro-reentrant LAT, the circuit was neighboring a reconnected PV in all but one. Thus, one may hypothesize that focal PV activity could be the trigger for reentry in these cases.

The fibrosis due to advanced atrial remodeling, as well as supplementary ablation lesions and, especially, gaps in the lesion sets, serves as the arrhythmogenic substrate for re-entry. It is well established that the rate and mechanism of LAT after PVI varies with the lesion set chosen for AF ablation, as well as the lesion quality [12,13,14]. Achieving a contiguous lesion set is the evident goal for durable PVI, and helps reduce the incidence of LAT, especially in normal LA. It is well known that handling these single shot devices is easier and requires less-experienced operators in comparison. Particularly for less-experienced operators, the ease of use of a single shot device may, therefore, help reduce the occurrence of LAT.

Our rate of LAT in the iRF group is in line with previous publications, citing rates of 2.9% after isolation of the pulmonary veins alone [15,16](see Table 4 for an overview of the current literature). For the cryoballoon, published data show rates of LAT of up to 11.3% [17]. In our population, iRF and cryoballoon revealed comparable rates of LAT, but the cryoballoon group comprised a higher percentage of patients with persistent AF. Further, in comparison, the cryoballoon patients with LAT showed lower rates of PV reconnection than the iRF group, leading to the assumption that the post cryoballoon LATs in the cryoballoon group were more likely to be substrate dependent.

Data on the lesion morphology created with the three studied ablation and energy technologies are scarce. The cryoballoon is known to lead to wider lesions, reaching further in the LA as compared to phased ablation. Magnetic resonance imaging studies comparing cryoballoon and RF ablation lesion contiguity are contradictory [21,22]. Cryoballoon lesions in a human autopsy case showed clearer demarcation compared to RF lesions [23,24]. Animal models showed similar findings [24]. As with all systems, the quality of the lesions is dictated by the tissue contact. The balloon-based system seems to struggle with the inferior parts of the veins.

Ablation with multielectrode catheters applying phased RF energy has been shown to create contiguous lesions in different animal models [25]. The circular multipolar design of the catheter seems to create easy, fast and effective PVI. However, the one year follow-up after PVI with this system revealed high rates of reconnection of multiple PVs in those with AF recurrence [6]. Similar findings have been reported in a comparison study of reconnection patterns with the cryoballoon [26]. The authors describe, for example, the reconnection of all four veins in 33% of the PVAC patients compared to 0% in the cryoballoon group, stipulating that our three groups showed comparable degrees of atrial substrate. The lowest rate of LAT after PVI with the PVAC system seems, therefore, surprising, but may be explained by the sample size.

Whether RF ablation using high-power short-duration (HPSD) or novel pulse field ablation technology reduces the rates of LAT remains to be determined. A comparative study in a pig model between conventional iRF and HPSD, with 90 W/4 s for PVI, showed HPSD to be superior to conventional iRF, in terms of lesion contiguity, transmurality, as well as uniformity [27]. The first data on pulsed field ablation show promising results in terms of acute and chronic lesion continuity and transmurality. Due to the non-thermal lesion formation, catheter–tissue contact is less important, which seems of particular interest in the quest for lower LAT rates and durable PVI [28,29].

## 5. Limitations

We present a non-randomized study; the choice of ablation catheter was made at the operator’s discretion. Further, we performed a non-continuous follow-up and may have missed some asymptomatic LATs, although, in our experience, LATs are, in general, symptomatic. Further, the use of newer developments in point-by-point RF ablation, such as high-power, short-duration and contact force sensing catheters, may have affected the LAT occurrence rate in the iRF group.

## 6. Conclusions

The occurrence of LAT after ablation of AF when limited to PVI only remains low, independent of the catheter technology or the energy source. The predominant mechanism of LAT was macro-reentrant tachycardia. In most patients with LAT, at least one PV was reconnected, which may play a role in LAT development. We did not find any predictors for LAT development.

## Figures and Tables

**Table 1:** Patient characteristics (NICM: non-ischemic cardiomyopathy).

	iRF (*n* = 300)	Cryo (*n* = 200)	PVAC (*n* = 150)	*p*-Value
Age at time of PVI (years)	58.9 ± 11	60.9 ± 11	58.4 ± 12	0.2
CHA_2_DS_2_-Vasc	1.79 ± 1.5	1.83 ± 1.5	1.89 ± 1.3	0.5
LVEF (%)	52.6 ± 11	61 ± 9	54.5 ± 11	0.5
Persistent AF (%)	32	45.5	33.1	0.05
Diabetes mellitus (%)	27	17	14	0.1
Arterial hypertension (%)	63	54.5	66	0.7
Male gender (%)	62.7	69.5	63.7	0.25
NICM (%)	3	8	2.7	0.45

**Table 2:** Patient characteristics of the LAT patients and mechanisms of LAT (EF: ejection fraction; AF: atrial fibrillation; LA: left atrial; CL: cycle length; LSPV: left superior pulmonary vein; LIPV: left inferior pulmonary vein; RIPV: right inferior pulmonary vein; RSPV: right superior pulmonary vein; RPV: right pulmonary veins; EP study: electrophysiological study).

Patient	PVI	EF	Gender	AF Type	LA Size	Flutter CL	PV	Mechanism of LAT
1	Cryo	65%	male	paroxysmal	53 mL/m^2^	280 ms	RSPV reconnected	Roof dependent
2	Cryo	63%	female	persistent	57 mL/m^2^	270 ms	N/A (No EP study)	N/A (No EP study)
3	Cryo	67%	female	paroxysmal	50 mL/m^2^	210 ms	RIPV reconnected	Microreentry RSPV
4	Cryo	52%	male	persistent	51 mL/m^2^	240 ms	N/A (No EP study)	N/A (No EP study)
5	Cryo	65%	male	persistent	19 mL/m^2^	220 ms	RSPV & RIPV reconnected	Mitralisthmus dependent
6	Cryo	56%	male	persistent	30 mL/m^2^	220 ms	N/A (No EP study)	N/A (No EP study)
7	Cryo	55%	female	paroxysmal	40 mL/m^2^	370 ms	No reconnection	Mitralisthmus dependent
8	Cryo	44%	male	persistent	36 mL/m^2^	240 ms	No reconnection	Macroreentry involving RPVs
9	PVAC	59%	Male	paroxysmal	40 mL/m^2^	250 ms	RSPV & RIPV reconnected	Mitralisthmus dependent
10	RF	60%	male	paroxysmal	N/A	245 ms	LSPV & LIPV reconnected	Mitralisthmus dependent
11	RF	50%	female	paroxysmal	25 mL/m^2^	270 ms	LSPV & RIPV reconnected	N/A (spontaneous LAT Termination)
12	RF	65%	female	paroxysmal	40 mL/m^2^	215 ms	LIPV & RSPV reconnected	Roof dependent
13	RF	59%	male	paroxysmal	29 mL/m^2^	280 ms	All 4 Veins reconnected	N/A (spontaneous LAT Termination)
14	RF	56%	male	persistent	36 mL/m^2^	250 ms	All 4 Veins reconnected	Microreentry posterior LSPV
15	RF	49%	female	persistent	34 mL/m^2^	240 ms	LSPV & LIPV reconnected	Roof dependent
16	RF	64%	female	paroxysmal	23 mL/m^2^	220 ms	No reconnection	Roof dependent
17	RF	58%	male	persistent	44 mL/m^2^	230 ms	LSPV& RSPV reconnected	Mitralisthmus dependent
18	RF	64%	male	paroxysmal	43 mL/m^2^	270 ms	RSPV reconnected	Multiple atrial tachycardia
19	RF	61%	male	paroxysmal	52 mL/m^2^	240 ms	No reconnection	Mitralisthmus dependent
20	RF	60%	male	persistent	58 mL/m^2^	280 ms	RSPV & RIPV reconnected	Microreentry posterior RSPV

**Table 3:** Univariate analysis of possible LAT predictors for the entire population, as well as the cryoballoon, iRF and PVAC populations (NICM: non-ischemic cardiomyopathy; ICM: ischemic cardiomyopathy; RF: radiofrequency; LSPV: left superior pulmonary vein; LIPV: left inferior pulmonary vein; RIPV: right inferior pulmonary vein; RSPV: right superior pulmonary vein; TTI: time to isolation).

	Entire Population	Entire Population	Entire Population	Cryoballoon	Cryoballoon	Cryoballoon	iRF	iRF	iRF	PVAC	PVAC	PVAC
	LAT (*n* = 20)	No LAT (*n* = 630)	*p*-Value	LAT (*n* = 8)	No LAT (*n* = 192)	*p*-Value	LAT (*n* = 11)	No LAT (*n* = 289)	*p*-Value	LAT (*n* = 1)	No LAT (*n* = 149)	*p*-Value
Age (years)	60.1 ± 12.8	59.4 ± 11	0.76	62 ± 12	61 ± 10	0.63	59.95 ± 13.59	58.84 ± 10.75	0.49	48	58.54 ± 11.72	0.3
Male Gender (*n*)	16	408	0.90	6	133	0.73	9	182	0.53	1	93	0.44
NICM (*n*)	2	27	0.39	1	15	0.6	1	8	0.44	0	4	0.87
ICM (*n*)	5	87	0.43	1	20	0.8	4	41	0.25	0	26	0.65
CHA_2_DS_2_-Vasc	2.2 ± 1.5	1.8 ± 1.4	0.1	1.63 ± 0.91	1.75 ± 1.3	0.9	2.44 ± 0.43	1.75 ± 0.1	0.10	3	1.89 ± 0.11	0.29
Ejection Fraction (%)	58.5 ± 11	55.4 ± 11	0.44	56.5 ± 11	56.11 ± 11	0.3	55.73 ± 9.65	51.6 ± 10.07	0.1	56	52.9 ± 1.78	0.92
Total RF Duration (min)	-	-	-	-	-	*-*	56.91 ± 29.68	51.69 ± 24.31	0.5	17	24.2 ± 10.2	0.34
Total Freeze Duration (s)	-	-	-	1552 ± 167	1399 ± 400	0.83	-	-	*-*	-	-	*-*
Total Number of Freezes	-	-	-	8.88 ± 1.88	8.08 ± 1.72	0.3	-	-	*-*	-	-	*-*
Mean of TTI LSPV (s)	-	-	-	50 ± 14	49 ± 22	0.73	-	-	*-*	-	-	*-*
Mean of TTI LIPV (s)	-	-	-	N.A.	62 ± 45	-	-	-	*-*	-	-	*-*
Mean of TTI RIPV (s)	-	-	-	120	53 ± 25	0.09	-	-	*-*	-	-	*-*
Mean of TTI RSPV (s)	-	-	-	60 ± 37	43 ± 18	0.63	-	-	*-*	-	-	*-*

**Table 4:** Exert of current literature on left atrial tachycardia after PVI [13,16,18,19,20].

Authors	Index PVI	FU Duration	LAT Rate
Ankerström et al.	iRF (*n* = 415) Cryoballoon (*n* = 215)	38 ± 21 months	4.8% 2.8%
Wasmer et al.	iRF (*n* = 839)	31 ± 17 months	4%
Deisenhofer et al.	iRF (*n* = 67)	NA	31%
Gerstenfeld et al.	iRF (*n* = 341)	NA	2.9%
Mikhaylov et al.	Cryoballoon (*n* = 181)	16 ± 93 months	8%

## Data Availability

The datasets used and/or analysed during the current study are available from the corresponding author on reasonable request.

## Supplementary Materials

The following supporting information can be downloaded at: https://www.mdpi.com/article/10.3390/jcdd9020050/s1, Video S1: Exemplary activation map of peri-mitral; Video S2: Exemplary activation map of roof-dependent flutters.

## References

[1] Williams B.A., Honushefsky A.M., Berger P.B. (2017). Temporal Trends in the Incidence, Prevalence, and Survival of Patients With Atrial Fibrillation From 2004 to 2016. Am. J. Cardiol..

[2] Kamel H., Okin P.M., Elkind M.S.V., Iadecola C. (2016). Atrial Fibrillation and Mechanisms of Stroke. Stroke.

[3] Wazni O.M., Dandamudi G., Sood N., Hoyt R., Tyler J., Durrani S., Niebauer M., Makati K., Halperin B., Gauri A. (2021). Cryoballoon Ablation as Initial Therapy for Atrial Fibrillation. N. Engl. J. Med..

[4] Kuck K.-H., Lebedev D.S., Mikhaylov E.N., Romanov A., Gellér L., Kalējs O., Neumann T., Davtyan K., On Y.K., Popov S. (2021). Catheter ablation or medical therapy to delay progression of atrial fibrillation: The randomized controlled atrial fibrillation progression trial (ATTEST). EP Eur..

[5] Kuck K.-H., Brugada J., Fürnkranz A., Metzner A., Ouyang F., Chun K.J., Elvan A., Arentz T., Bestehorn K., Pocock S.J. (2016). Cryoballoon or Radiofrequency Ablation for Paroxysmal Atrial Fibrillation. N. Engl. J. Med..

[6] Spitzer S.G., Leitz P., Langbein A., Karolyi L., Scharfe F., Weinmann T., Rämmler C., Pott C., Mönnig G., Eckardt L. (2017). Circumferential pulmonary vein isolation with second-generation multipolar catheter in patients with paroxysmal or persistent atrial fibrillation: Procedural and one-year follow-up results. Int. J. Cardiol..

[7] Bittner A., Mönnig G., Zellerhoff S., Pott C., Köbe J., Dechering D., Milberg P., Wasmer K., Eckardt L. (2011). Randomized study comparing duty-cycled bipolar and unipolar radiofrequency with point-by-point ablation in pulmonary vein isolation. Heart Rhythm.

[8] Leitz P., Güner F., Wasmer K., Foraita P., Pott C., Dechering D.G., Zellerhoff S., Kochhäuser S., Lange P.S., Eckardt L. (2015). Data on procedural handling and complications of pulmonary vein isolation using the pulmonary vein ablation catheter GOLD^®^. Europace.

[9] Wasmer K., Krüsemann D., Leitz P., Güner F., Pott C., Zellerhoff S., Dechering D., Köbe J., Lange P.S., Eckardt L. (2016). Lower rate of left atrial tachycardia after pulmonary vein isolation with PVAC versus irrigated-tip circumferential antral ablation. Heart Rhythm.

[10] Leitz P., Mönnig G., Güner F., Dechering D.G., Wasmer K., Reinke F., Lange P.S., Eckardt L., Frommeyer G. (2018). Comparing learning curves of two established “single-shot” devices for ablation of atrial fibrillation. J. Interv. Card. Electrophysiol..

[11] Pathak R., Lau D.H., Mahajan R., Sanders P. (2013). Structural and Functional Remodeling of the Left Atrium: Clinical and Therapeutic Implications for Atrial Fibrillation. J. Atr. Fibrillation.

[12] Hashimoto K., Watanabe I., Kofune M., Ashino S., Okumura Y., Ohkubo K., Shindo A., Sugimura H., Nakai T., Saito S. (2005). Left Atrial Tachycardia After Pulmonary Vein Isolation for Atrial Fibrillation. J. Arrhythmia.

[13] Akerström F., Bastani H., Insulander P., Schwieler J., Arias M.A., Jensen-Urstad M. (2014). Comparison of Regular Atrial Tachycardia Incidence After Circumferential Radiofrequency versus Cryoballoon Pulmonary Vein Isolation in Real-Life Practice. J. Cardiovasc. Electrophysiol..

[14] Oral H., Knight B.P., Morady F. (2003). Left atrial flutter after segmental ostial radiofrequency catheter ablation for pulmonary vein isolation. Pacing Clin. Electrophysiol..

[15] Oral H., Knight B.P., Tada H., Özaydın M., Chugh A., Hassan S., Scharf C., Lai S.W., Greenstein R., Pelosi F. (2002). Pulmonary Vein Isolation for Paroxysmal and Persistent Atrial Fibrillation. Circulation.

[16] Gerstenfeld E.P., Callans D.J., Dixit S., Russo A.M., Nayak H., Lin D., Pulliam W., Siddique S., Marchlinski F. (2004). Mechanisms of Organized Left Atrial Tachycardias Occurring After Pulmonary Vein Isolation. Circulation.

[17] Lyan E., Yalin K., Abdin A., Sawan N., Liosis S., Lange S.A., Eitel I., Heeger C.-H., Meyer-Saraei R., Eitel C. (2019). Mechanism, underlying substrate and predictors of atrial tachycardia following atrial fibrillation ablation using the second-generation cryoballoon. J. Cardiol..

[18] Wasmer K., Mönnig G., Bittner A., Dechering D., Zellerhoff S., Milberg P., Köbe J., Eckardt L. (2012). Incidence, characteristics, and outcome of left atrial tachycardias after circumferential antral ablation of atrial fibrillation. Heart Rhythm.

[19] Deisenhofer I., Estner H., Zrenner B., Schreieck J., Weyerbrock S., Hessling G., Scharf K., Karch M.R., Schmitt C. (2006). Left atrial tachycardia after circumferential pulmonary vein ablation for atrial fibrillation: Incidence, electrophysiological characteristics, and results of radiofrequency ablation. Europace.

[20] Mikhaylov E.N., Bhagwandien R., Janse P.A., Theuns D.A., Szili-Torok T. (2013). Regular atrial tachycardias developing after cryoballoon pulmonary vein isolation: Incidence, characteristics, and predictors. Europace.

[21] Kurose J., Kiuchi K., Fukuzawa K., Takami M., Mori S., Suehiro H., Nagamatsu Y., Akita T., Takemoto M., Yatomi A. (2020). Lesion characteristics between cryoballoon ablation and radiofrequency ablation with a contact force-sensing catheter: Late-gadolinium enhancement magnetic resonance imaging assessment. J. Cardiovasc. Electrophysiol..

[22] Kurose J., Kiuchi K., Fukuzawa K., Mori S., Ichibori H., Konishi H., Taniguchi Y., Hyogo K., Imada H., Suehiro H. (2018). The lesion characteristics assessed by LGE-MRI after the cryoballoon ablation and conventional radiofrequency ablation. J. Arrhythmia.

[23] Hirao T., Nitta J., Adachi A., Takahashi Y., Goya M., Hirao K. (2018). First confirmation of histologic changes in the human heart after cryoballoon ablation. HeartRhythm Case Rep..

[24] Andrade J., Dubuc M., Guerra P.G., Landry E., Coulombe N., LeDuc H., Rivard L., Macle L., Thibault B., Talajic M. (2013). Pulmonary Vein Isolation Using a Second-Generation Cryoballoon Catheter: A Randomized Comparison of Ablation Duration and Method of Deflation. J. Cardiovasc. Electrophysiol..

[25] Hocini M., Condie C., Stewart M.T., Kirchhof N., Foell J.D. (2016). Predictability of lesion durability for AF ablation using phased radiofrequency: Power, temperature, and duration impact creation of transmural lesions. Heart Rhythm.

[26] Wieczorek M., Sassani K., Hoeltgen R. (2020). Comparison of pulmonary vein reconnection patterns after multielectrode phased radiofrequency- and cryoballoon ablation of atrial fibrillation. BMC Cardiovasc. Disord..

[27] Leshem E., Zilberman I., Tschabrunn C.M., Barkagan M., Contreras-Valdes F.M., Govari A., Anter E. (2018). High-Power and Short-Duration Ablation for Pulmonary Vein Isolation. JACC Clin. Electrophysiol..

[28] Do C.J.B., Haines D.E. (2020). Pulsed field ablation for pulmonary vein isolation in the treatment of atrial fibrillation. J. Cardiovasc. Electrophysiol..

[29] Stewart M.T., Haines D.E., Miklavčič D., Kos B., Kirchhof N., Barka N., Mattison L., Martien M., Onal B., Howard B. (2021). Safety and chronic lesion characterization of pulsed field ablation in a Porcine model. J. Cardiovasc. Electrophysiol..

